# Th1^high^ in tumor microenvironment is an indicator of poor prognosis for patients with NSCLC

**DOI:** 10.18632/oncotarget.14471

**Published:** 2017-01-03

**Authors:** Huang Jian, Shen Fangrong, Huang Haitao, Ling Chunhua, Zhang Guangbo

**Affiliations:** ^1^ Department of respiratory, The First Affiliated Hospital of Soochow University, Suzhou, 215007, China; ^2^ Department of Emergency, The First Affiliated Hospital of Soochow University, Suzhou, 215007, China; ^3^ Clinical Immunology Laboratory, The First Affiliated Hospital of Soochow University, Suzhou, 215007, China; ^4^ Department of Thoracic Surgery, The First Affiliated Hospital of Soochow University, Suzhou, 215007, China

**Keywords:** Th subset, prognosis, tumor microenvironment, NSCLC

## Abstract

CD4+Th subsets play an important role in tumor progression but their expression characteristics and clinical significance in human tumor microenvironment remains unclear. In this study, we aim to analyze the expression and clinical significance of tissue-infiltrating Th1, Th2 and Th17 in lung cancer by flow cytometry. We found that the frequency of CD3^+^CD4^+^IFN-γ^+^Th1 in tumor nest was significantly lower than that in tumor boundary, adjacent normal lung tissue or corresponding lymph node tissue; the frequency of CD3^+^CD4^+^IL-4^+^Th2 in tumor nest was significantly higher than that in tumor boundary, adjacent normal lung tissue or corresponding lymph node tissue; the frequency of CD3^+^CD4^+^IL-17^+^Th17 in tumor nest was significantly lower than that in tumor boundary, but not adjacent normal tissue or corresponding lymph node tissue. Survival analysis of 2-years survival after surgery showed that Th1^high^ group was significantly lower compared with Th1^low^ group; Th2^high^ and Th17^low^ is a good prognosis index compared with the Th2^low^ and Th17^high^ groups respectively, but this difference failed to significance. In addition, we also found that PD-1 expression showed a high level on lung tumor tissues and adjacent non- tumor tissue infiltrating T cells, and no significant difference was found between the two groups. However PD-L1 on CD45^+^CD14^+^mononcytes/macrophages in tumor tissue show a significantly higher level compared with that in adjacent nontumor tissues. *In vitro* stimulation experiments showed that IFN-γ could significantly increase PD-L1 expression on monocyte. In conclusion, we for the first time found Th1^high^ is a poor indicator for prognosis of lung cancer.

## INTRODUCTION

Lung cancer is a malignant disease that associated with the highest mortality rate (18.2%) of all types of cancer worldwide [1]. Solid tumors from the occurrence to clinical findings generally have to go through more than ten years or even longer. In the process of tumorigenesis, a suitable microenvironment for tumor growth gradually formed in tissue microenvironment [1–3]. Therefore, it is very important to understand the characteristics of the microenvironment for investigation of the occurrence and development of tumor *in vivo*.

Tumor microenvironment is composed of different types of stromal cells. In fact, as early as 1880s, Steven Paget proposed the “seed and soil” hypothesis, suggesting that a fertile “soil” (the microenvironment) is essential for the “seed” (the tumor cells) to grow. Stromal cells promote the growth of tumor cells by secreting growth factors, chemokines and inflammatory factors [4]. Immune cells are important stromal cells in the tumor microenvironment, mainly including T lymphocytes and myeloid derived cells [1]. Myeloid derived cells are mainly monocytes/macrophages, dendritic cells and a group of newly discovered myeloid derived suppressor cells (MDSC). T cells mainly include cytotoxic T cells and helper T cells. The helper T cells were mainly divided into Th1, Th2, Th9, Th17, and Th22 according to the cytokine-secreting character. It is generally believed that Th1 is the main anti-tumor reactive T cells, and Th2 plays an important role in promoting tumor immune escape [2]. Th17 plays an important role in the inflammatory response, but in recent years, it is found that Th17 also could promote the development of tumor by affecting the tumor angiogenesis [5–7]. A little of knowledge about Th9 and Th22 in the tumor research was reported, and there is no clear conclusion.

According to the definition, Th subsets detection needed analyze CD4 combined with specific cytokines such as CD4^+^IFN-γ^+^ for Th1, CD4^+^IL-4^+^ for Th2, and CD4^+^IL-17^+^ for Th17. Typically, T cells after stimulation by ionomycin combined with phorbol myristate acetate (PMA) were collected for th subsets analysis by flow cytometry. So, fresh cells are necessary for detection of Th subsets in tumor microenviroments. Most of previous studies have focused on the expression and clinical significance of helper T cells in peripheral blood of cancer patients, but the study of Th subsets in human tumor tissue infiltration is little using flow cytometry [8–11]. Tosolini et al reported that tissue infiltrating Th subsets in patient with colon cancer, but the method used is immunohistochemistry (IHC), which might be not accurate enough to quantify the levels of Th subsets compared with flow cytometry.

In conclusion, the expression characteristics and clinical significance of Th subsets in human tumor tissues are still poorly understood. In this study, we analyzed the expression characteristics and clinical significance of Th1, Th2 and Th17 in different anatomical sites of lung cancer using flow cytometry.

## RESULTS

### The detection scheme of Th subsets in tumor tissue environment was established

**Figure 1:**
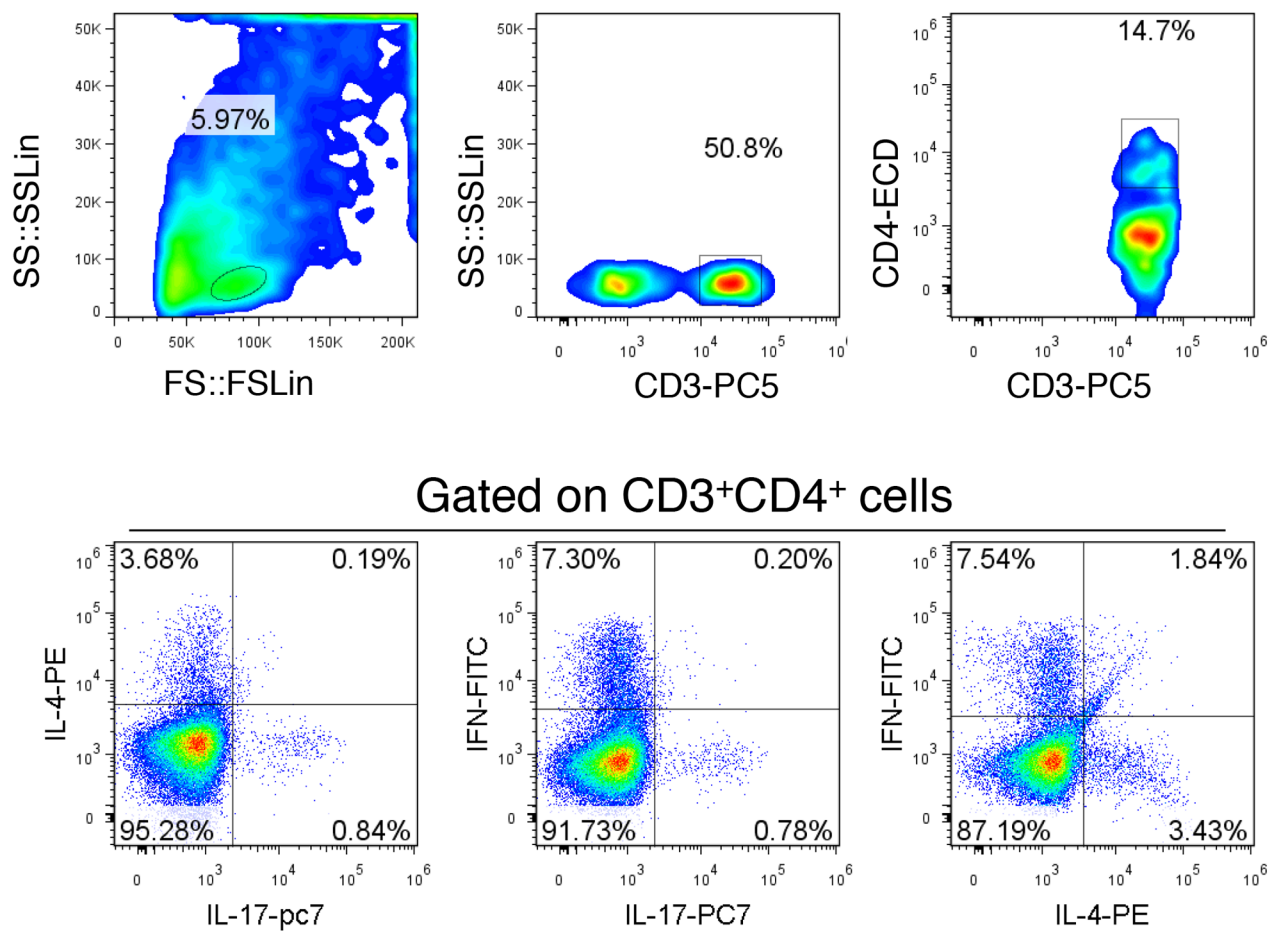
Scheme of the Th subgroup detection The lymph node tissue was selected to establish detection scheme. Gated on CD3+CD4+ cells were co-labeled with IFN-γ, IL-4 and IL-17. The results show that there is little cross reaction between each other, but the distinction was still relatively ideal. This is a representative result of three independent experiments.

As shown in Figure 1, Th1 cells were found that can be identified as CD3^+^CD4^+^IFN-γ^+^ IL-4^-^IL-17^-^; Th1 cells were found that can be identified as CD3^+^CD4^+^IL-4^+^IFN-γ^-^IL-17^-^; Th17 cells were found that can be identified as CD3^+^CD4^+^IL-4^-^IFN-γ^-^IL-17^+^. These results indicate that the three cross identification is little, so CD3^+^CD4^+^IFN-γ^+^ can be identified as Th1 cells; CD3^+^CD4^+^IL-4^+^ cells can be identified as Th2 cells; CD3^+^CD4^+^IL-17^+^ cells can be identified as Th17 cells.

### Th1 decreased in tumor nest

**Figure 2:**
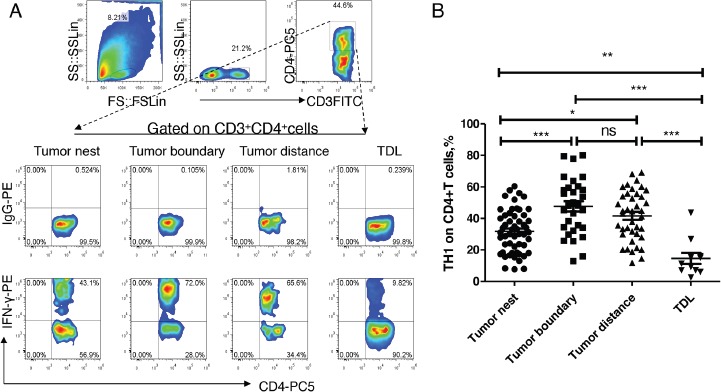
Expression and distribution of Th1 subsets in lung cancer microenvironment **A.** different tissue-infiltrating Th1 subset detected by flow cytometry; **B.** data statistics of Th1 detected by flow cytometry, data is represented as mean±SEM, each dot represents a sample. Statistical method is Kruskal-Wallis test. * * represents p<0.01, * represents <0.05.

Firstly, we analyzed the distribution of Th1 levels in lung tissue at different locations and regional lymph nodes from patients with NSCLC. As shown in Figure 2, the median levels of Th1 in tumor nest tissue (n =54) was 31.14%, and mean levels were 31.81±1.85%; the median levels of Th1 in tumor-normal junction tissue (n =30) was 48.18%, and mean levels were 47.62±3.28%; the median levels of Th1 in normal lung tissues (n =40) was 43.85%, and mean levels were 41.64±2.49%; the median levels of Th1 in tumor-regional lymph nodes (n =11) was 11.26%, and mean levels were 14.61±3.53%. Comparative analysis showed that there were significant differences between each other (P< 0.01). Paired comparisons showed that Th1 levels in carcinoma tissue was significantly lower than that in tumor-normal junction tissue and that in adjacent normal tissues (P <0.05, respectively), but was significantly higher than that in lymph node tissues (P <0.05). There was no significant difference of Th1 levels between the adjacent normal tissues and tumor-normal junction tissue (P >0.05). Additionally, Th1 levels of lymph node tissue were significantly lower than that of anyone of the three groups (P <0.05).

### Th2 increased in tumor nest

**Figure 3:**
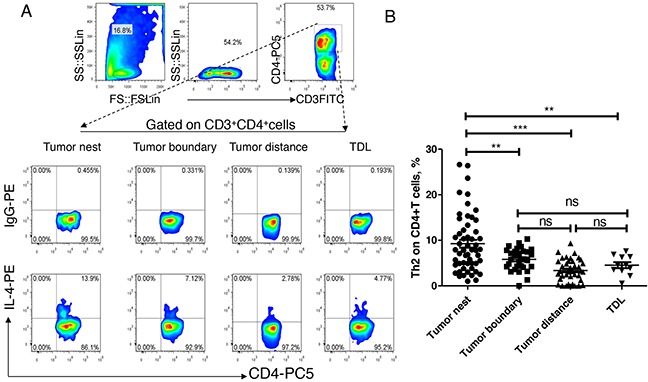
Expression and distribution of Th1 subsets in lung cancer microenvironment **A.** different tissue-infiltrating Th2 subset detected by flow cytometry; **B.** data statistics of Th2 detected by flow cytometry, data is represented as mean±SEM, each dot represents a sample. Statistical method is Kruskal-Wallis test. * * represents p<0.01, * represents <0.05.

Secondly, we analyzed the distribution of Th2 levels in above referred tissues. As shown in Figure 3, the median levels of Th2 in tumor nest tissue (n =54) was 7.855%, and mean levels were 9.25±0.86%; the median levels of Th2 in tumor-normal junction tissue (n =30) was 5.82%%, and mean levels were 5.80±0.45%; the median levels of Th2 in normal lung tissues (n =40) was 3.20%, and mean levels were 3.37±0.39%; the median levels of Th2 in tumor-regional lymph nodes (n =11) was 4.50%, and mean levels were 4.54±0.65%. Comparative analysis showed that there were significant differences between each other (P< 0.01). Paired comparisons showed that Th2 level in carcinoma tissue was significantly higher than that of anyone of the other three groups (P<0.05, respectively). There was no significant difference of Th2 levels between each other of the adjacent normal tissues, tumor-normal junction tissue and lymph node tissue (P>0.05).

### Th17 increased in cancerous peripheral tissue

**Figure 4:**
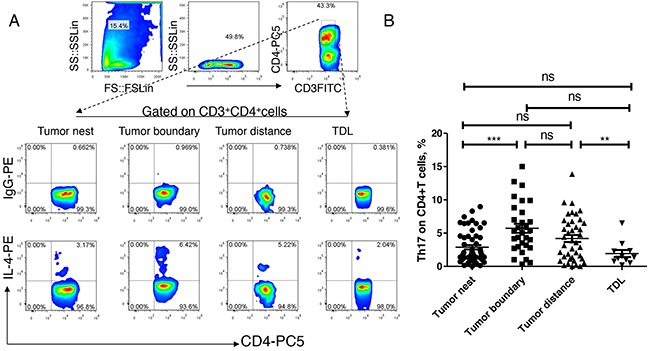
Expression and distribution of Th17 subsets in lung cancer microenvironment **A.** different tissue-infiltrating Th17 subset detected by flow cytometry; **B.** data statistics of Th17 detected by flow cytometry, data is represented as mean±SEM, each dot represents a sample. Statistical method is Kruskal-Wallis test. * * represents p<0.01, * represents <0.05.

Finally, we analyzed the expression and distribution of Th17 in lung cancer. As shown in Figure 4, the median levels of Th17 in tumor nest tissue was 1.86%, and mean levels were 2.87 ±0.32%; the median levels of Th17 in tumor-normal junction tissue (n =30) was 5.15%, and mean levels were 5.73 ±0.69%; the median levels of Th2 in normal lung tissues (n =40) was 3.74%, and mean levels were 4.19 ±0.53%; the median levels of Th17 in tumor regional lymph node (n =11) was 1.40%, the mean score was 1.93 ±0.53%. Comparative analysis showed that there was no significant difference among the four groups (P> 0.05). Paired comparisons showed that the levels of Th17 in the tumor tissue was significantly lower than that in tumor-normal junction tissue, and the Th17 was significantly higher in normal lung tissues than that in the regional lymph nodes. There was no significant difference among the other groups (P> 0.05).

### Clinical significance of expression of Th1, Th2 and Th17 in lung cancer tissues

To explore the clinical significance of Th subsets in lung cancer, we analyzed the expression levels of tumor-nest infiltrating Th1, Th2 and Th17, and the clinical parameters. The results are shown in Table 1, no significant correlation was found between the level of Th1 and age, gender, tumor size, pathological type, lymph node metastasis or clinical stage of tumor; only one significant correlation between the expression level of Th2 and the pathological types: Th2 level in squamous cell carcinoma were significantly lower than those in other types; Additionally, the level of Th17 in patients with age=<60 years old was significant lower than that in patients with age>60 years old.

**Table 1:** Clnical significance of Th1, Th2 and Th17 in progression of NSCLC (n=54)

Character	Th1		p	Th2		p	Th17		p
	low	high		low	high		low	high	
**Sex**									
Female	9	8	1	7	10	0.559	10	7	0.559
Male	18	19		20	17		17	20	
**Age,y**									
<60	10	10	1	10	10	1	14	6	0.047*
>=60	17	17		17	17		13	21	
**Histologic subtype**									
Ad	8	13	0.353	10	11	0.023**	10	11	0.25
SCC	11	9		14	6		8	12	
Others	8	5		3	10		9	4	
**Tumor size,cm**									
<=3	6	5	1	6	5	1	5	6	1
>3	21	22		21	22		22	21	
**T factor**									
1&2	21	15	0.148	17	19	0.733	18	18	1
3&4	6	12		10	8		9	9	
**N factor**									
0	12	12	0.711	11	13	0.309	9	15	0.159
1	5	3		6	2		6	2	
2	10	12		10	12		12	10	
**Tumor stage**									
I & II	15	11	0.414	14	12	0.7857	12	14	0.786
III & IV	12	16		13	15		15	13	

** represents p<0.01; * represents <0.05.

Ad represents Adenocarcinoma; SCC represents Squamous cell carcinoma.

### Th1^low^ is a good prognostic indicator in lung cancer tissue

**Figure 5:**
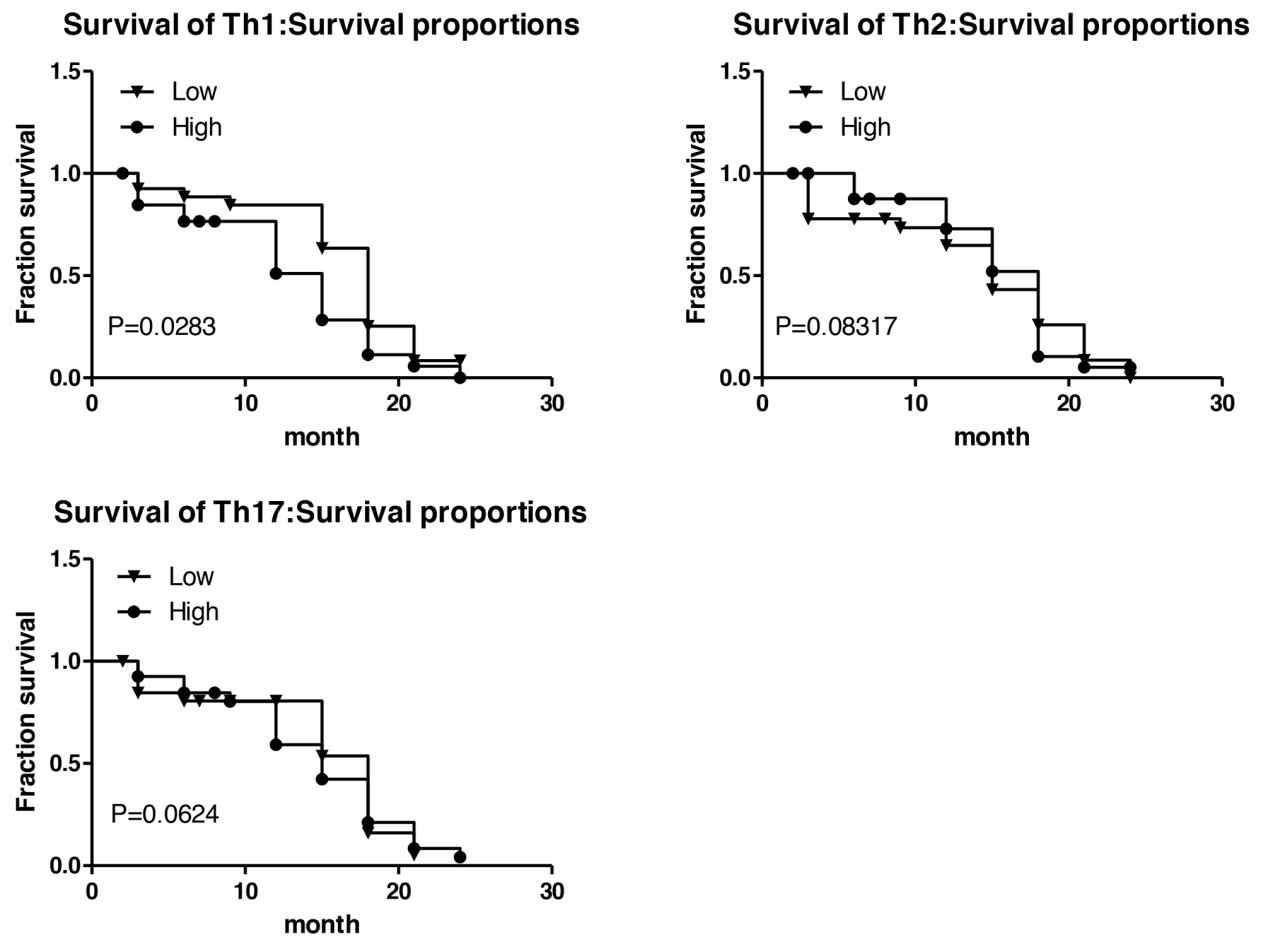
Survival time associated with Th1, Th2, Th17 subsets **A.** The relationship between Th1 and survival of patients with lung cancer; **B.** The relationship between Th2 and survival of patients with lung cancer; **C.** The relationship between Th17 and survival of patients with lung cancer. The Th subsets were divided into low and high group according to the corresponding median level. Log-rank (Mantel-Cox) Test was performed as statistical method.

In order to explore the value of Th subsets in the clinical prognosis of lung cancer, 54 patients, who had received surgical operation with 2-year follow-up data available, were further subjected to survival analysis. The Th subsets in tumor nests were divided into low and high group according to the corresponding median level. The results are shown as Figure 5, Th1^low^ group had a significantly longer recurrence-free survival time than Th1^high^ group (P<0.05); Th2^low^ group had a shorter recurrence-free survival than Th2^high^ group, indicating Th2^high^ shows a biological indicator of good prognosis, but there is no significant differences (P>0.05); Th17^low^ had a shorter recurrence-free survival than Th17^high^ group, but still no significant differences were found (P>0.05). These results suggested that the frequency of Th1 subset correlated with progression of NSCLC, and Th1^high^ might serve as a poor prognostic marker for the survival of patients with NSCLC.

### IFN-γ can significantly enhance the PD-1/PD-L1 signal in tumor microenvironment

**Figure 6:**
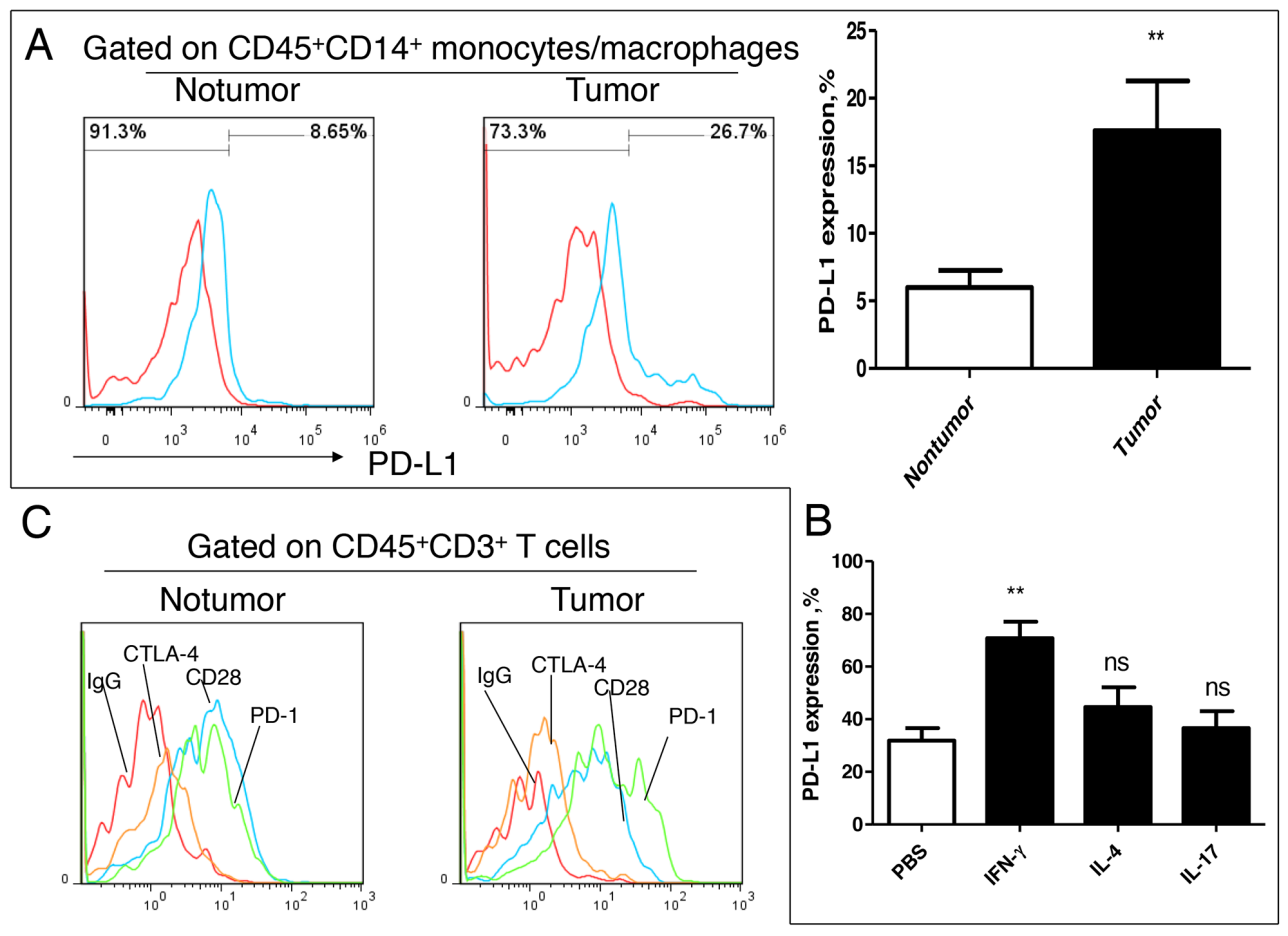
Effect of IFN-γ on PD-1/PD-L1 signal in lung cancer microenvironment **A.** PD-L1 expression analysis on tissue infiltration of mononcyte/macrophage from patients with NSCLC (n=6); **B.** Effect of exogenous cytokines IL-4, IL-17 and IFN-γ on expression of PD-L1 on monocyte/macrophage (n=6); **C.** Expression analysis Immune checkpoint molecule PD-1, CD28 and CTLA-4 on infiltrating T cell in lung cancer tissues. This is a representative result of three independent experiments.

In order to explore the effects of Th1 subset on the immune microenvironment, we analyzed the expression of immune receptor molecules on T lymphocytes infiltrating lymphocytes in lung cancer. We firstly analyzed the expression of PD-L1 in the infiltrating mononcytes-macrophages, and we found that PD-L1 levels in tumor tissues were significantly higher comparing with those in nontumor tissues (Figure 6A). *In vitro* analysis, we found that IFN-γ rather than IL-4 and IL-17 could significantly induce PD-L1 expression on monocytes (Figure 6B). At the same time, we also found that there were high expression of PD-1 and CD28, and low expression of CTLA-4 on T cells in cancer tissues and in distal nontumor tissues (Figure 6C). Comparative analysis showed that the PD-1 level was significantly higher than that of CD28 in the tumor tissues but not in nontumor tissues (Figure 6C). These results suggested that Th1^high^ may enhance the PD-1/PD-L1 signal by secreting a higher level of IFN-γ, and promote the formation of the microenvironment of tumor escape

## DISCUSSION

Immune status determines the carcinogenesis. T lymphocytes, including helper T cells (Th) and cytotoxic T cells (Tc), are important components of the immune system in tumor microenvironment, which participate in tumor progression through immune regulation. According to the function and phenotype, Th subsets are mainly divided into Th1, Th2, Th9, Th17, Th22, Thf and Treg [13–19]. Among them, Th1, Th2, Th17 and Treg are more concerned in tumor immunity.

It is usually considered that Th1 enhances tumor immune surveillance of tumor; Th2 and Treg are associated with the tumor immune evasion. With development of tumor *in vivo*, the level of Th1 decline, along with the proportion of Th2 and Treg gradually increase, which formats tumor immune microenvironment [20–23]. Th17 was initially thought to be anti tumor immune cells, but subsequent studies to prove that Th17 can promote tumor angiogenesis in the promotion of tumor progression [24]. Therefore, Th1^high^Th2^low^Th17^low^Treg^low^ should be the characteristic of immunity for anti-tumor in microenvironment, and is also an immune state index to inhibit tumor progression. However, the understanding about the distribution of Th1, Th2, and Th17 and Treg in the human solid tumor tissue environment remains unclear, and its clinical significance is still lack of solid evidence. Due to method is different from Th1, Th2 and Th17, and the limited cell numbers were considered, Treg was not analyzed in this study.

In this study, the distribution of Th1, Th2 and Th17 were detected by flow cytometry. The method was used to overcome the limitation of the single index of immunohistochemistry, and the quantitative analysis was more accurate. Secondly, this study collected tissues at the different anatomical sites of the patients, and provided a little of knowledge to understand the distribution of Th subsets in lung cancer.

We firstly analyzed tissue distribution of Th1, Th2 and Th17 in tumor microenvironment. Compared with the distant normal tissue and the adjacent tumor tissues, the Th1 subsets significantly decreased in tumor nest tissues, suggesting that the Th1 type immune response was weakened in tumor sites. But the Th1 level was still significantly higher in the tumor nest compared with that in regional lymph node, which suggested that the Th1 response still remain a higher level than that in periphery. That is to say that the Th1 immune response in cancer nest shows significantly lower levels than that in the peritumor; but even weakened, Th1 responses in tumor nest still shows a significantly higher level compared with that in regional lymph node. As far as Th2 is concerned, the highest levels were found in tumor nest, suggesting that the Th2 type response was the highest in tumor region than the other peritumor parts including adjacent tumor tissues, distant normal tissues and regional lymph node. However, the distribution of Th17 is different from Th1 and Th2. Th17 subgroup was significantly lower in the cancer region than that in peritumor regions. Th17 can inhibit the apoptosis of myeloid derived cells as well as promoting the chemotactical accumulation of myeloid derived cells. It has a good explanation of the most abundant of myeloid derived cells in tumor-nontumor junction area, and the angiogenesis is more active. The importance of myeloid derived cells to participate in angiogenesis has been widely accepted.

But we also got some unexpected results. It is generally considered that Th1^high^Th2^low^ is a good indicator for anti-tumor. However, we found that the survival time of Th1^low^ group was significantly longer than that of Th1^high^ group, which suggested that Th1^high^ was a novel indicator of poor prognosis. In contrast, lung cancer survival in Th2^high^ group was shorter than that in the Th2^low^ group, but no significant difference was found between Th2^high^ and Th2^low^ groups. Recently, the role of Th17 in cancer has been focused widely [24, 25]. In this study, we found that the level of Th17 in cancer was lower than that in cancer and cancer, although it did not reach significant difference.

It is generally believed that IFN-γ can activate macrophages and NK cells, which playing an important role in anti-tumor [26]. However, previous paper also reported that IFN-γ can promote tumor progression [27–29]. Xiao et al reported that IFN-γ can promote skin inflammatory reaction mediated by IL-17 in promoting tumor growth [31]. Medina-Echeverz et al reported that IFN-γ regulates survival of granulocytic MDSCs by STAT1-dependent pathway [32]. Therefore, Th1^high^ is a good indicator of tumor prognosis is also possible, but the specific reasons needed further studies. In this study, we found that IFN-γ could enhance the PD-1/PD-L1 signal in the tumor microenvironment. In recent years, the PD-1/PD-L1 immune checkpoint treatment against tumor has made great progress, which indicating PD-1/PD-L1 signal for tumor immune escape is very important. IFN-γ has an antitumor effect, but clinical trials of IFN-γ in tumor treatment did not achieve the desired results, which may be resulted from the increase of PD-L1 induced on macrophages and tumor cells by IFN-γ. Therefore, the combination of IFN-γ with PD-1/PD-L1 blocking mAb may be a new strategy for tumor treatment.

In conclusion, this study systematically investigated the expression and clinical significance of Th1, Th2 and Th17 in lung cancer tissues. We for the first time found that Th1^high^ is a poor prognostic biomarker for lung cancer. Although biological role or mechanism needs a lot of work, this study provides a new perspective for understanding the role of Th subsets in the development and treatment of lung cancer.

## MATERIALS AND METHODS

### General information of patients

A total of 54 patients with lung cancer were collected in this study. These patients were diagnosed with pathology and underwent a primary resection at the First Hospital Affiliated to Suzhou University. Specimens were collected from November 2009 to July 2011. 17 cases of female; 37 cases were male; median age was 63 years, the range is 42-77 years; 20 cases of squamous cell carcinoma, 21 cases of adenocarcinoma, and other types with a total of 13 cases including large cell cancer (5 cases), alveolar cell carcinoma (4 cases), mucinous adenocarcinoma (2 cases) and mixed type of cancer (2 cases). Lobectomy with radical mediastinal and hilar lymphadenectomy was performed on all patients. Cancer staging was based on TNM of the International Union against Cancer. 2-year survival time was defined as the time period from the date of surgery to the confirmed dead date of patients of last follow-up. This study was approved by the ethics committee of First Hospital Affiliated to Suzhou University.

### Sampling and sample handling

Tumor tissues were taken from areas of solid tumor tissues lacking the gross aspect of massive necrosis. The tumor-free normal lung tissue samples were taken at least 5 cm away from the tumor margin; the tumor-boundary lung tissue samples were taken at most 2 cm away from the tumor margin. Lymph node is the tumor area surgery to clean the lymph nodes. All of the above samples were obtained under the guidance of the pathology department, without affecting the pathological diagnosis. Fresh tumors, corresponding normal tissues and Lymph node were all used for the isolation of tissue-infiltrating leukocytes. Collection of single cell suspension was performed according to our previous paper [12]. In brief, after washing with PBS to remove blood cells, tissues were cut into pieces and moved into the containing medium with 0.1% collagenase IV (sigma) + 5% calf serum and culture for 60 mins at 37°C; 20% bovine serum 1640 culture medium was used to terminate the digestion reaction; Samples were grinded, and passed through a 150-μm mesh to remove tissue fragments and thereafter through a 30-μm filter (Miltenyi biotec) to remove cell clusters. The obtained single cell suspension was performed for analysis by flow cytometry.

### Flow cytometry analysis

single cell suspension was treated with PMA (1 μg/ml) and Ionomycin (1μg/ml) for 1 hr at 37°C, After that BFA (1:1000) additionally added and then continue to stimulated for 4-5hr at 37°C. After stimulation, cells were collected for antibody staining. Th1 is defined as the CD3^+^CD4^+^IFN-γ^+^subgroup; Th2 is defined as the CD3^+^CD4^+^IL-4^+^ subgroup; Th17 is defined as the CD3^+^CD4^+^IL-17^+^ subgroup. Th (%)=Th subgroup/CD3^+^CD4^+^ subgroup x 100%.

### PD-1/PD-L1 expression analysis *in vitro*

To assess PD-L1 expression by Th cytokines, we first isolated PBMCs were purified by StemSep™ Human CD14 Positive Selection Kit (Catalog # 14758, STEMCELL Technologies, Canada). The purified monocytes were then treated with PBS control, IL-17 (Accession #: Q9H293, R&D Systems, USA), IFN-γ (Accession #: CAA31639, R&D Systems, USA) or IL-4 (Accession #: P05112, R&D Systems, USA). After a 24 hr culture, the stimulated monocytes were subjected to flow cytometric analysis of PD-L1 levels.

### Statistical methods

Statistical analysis was done with GraphPad Prism 5 software (version 5.5). Statistical analysis for normally distributed values was performed using Student's *t* test or ANOVA. Non-normally distributed values, as assessed by the Kolmogorov-Smirnov test, were analyzed by the Mann-Whitney *U* test. Clinical parameters and th correlation was analyzed using chi square or Fisher's exact test; survival analysis for log-rank (Mantel-Cox) test. Data expressed as mean + SEM. The *P* value <0.05 was considered as statistical significance.
