# Correction: Th1^high^ in tumor microenvironment is an indicator of poor prognosis for patients with NSCLC

**DOI:** 10.18632/oncotarget.18440

**Published:** 2017-06-12

**Authors:** Huang Jian, Shen Fangrong, Huang Haitao, Ling Chunhua, Zhang Guangbo

**Present**: Author contributions were not correctly attributed..

**Correct:** The proper author contributions are shown below.

Huang Jian and Shen Fangrong have contributed to this work and are co-first authors.

Original article: Oncotarget. 2017; 8:13116-13125. doi: 10.18632/oncotarget.14471

